# Beyond Structure-Function: Getting at Sustainability within Biomimicry Pedagogy

**DOI:** 10.3390/biomimetics7030090

**Published:** 2022-07-06

**Authors:** Benjamin Linder, Jean Huang

**Affiliations:** Olin College of Engineering, Needham, MA 02446, USA; jhuang@olin.edu

**Keywords:** biomimicry, conditions conducive to life, pedagogy, conceptual scaffolding, structure-function, sustainability

## Abstract

Biomimicry practice and pedagogy unify biology and design for problem solving inspired by nature. Pedagogy that supports biomimicry practice can facilitate the development of novel solutions to address societal needs and challenges. Even though biomimicry affords the possibility to address sustainability, its current practice does not necessarily lead to doing so, which can result in exploitation of nature and increased unsustainability. Recognition of this risk exists but is not yet widespread in biomimicry pedagogy, and few structured methodologies are available to support learner’s efforts towards sustainability. The difficulties associated with incorporating sustainability within biomimicry are numerous and varied. In this report, we contribute to an understanding of incorporating sustainability in teaching and learning. We describe a pedagogical framing and conceptual scaffolding developed and used to bring sustainability into a biomimicry course for design- and biology-minded engineering students that integrates available biomimicry and design language, tools, and methods. We scaffold consideration of structure-function and conditions conducive to life separately, and then unify these perspectives in a way that is accessible to students. This approach centralizes sustainability in biomimicry practice and asks students to consider the ethics of design practice and responsibility to the natural world. We are encouraged by student outcomes, observing clear signs of creative systemic thinking and higher-level learning from nature. Based on pre- and post-design sprint results, students significantly shifted away from a narrower structure-function practice towards addressing conditions conducive to life. We propose that biomimicry educators and facilitators make a commitment to always include a sustainability approach within their pedagogy or explicitly acknowledge their delivery does not provide for it.

## 1. Introduction

Our interest lies with pedagogy for addressing sustainability within biomimicry practice. Biomimicry is an expansive multi-disciplinary design practice spanning nature to innovation: everything from insights into materials and mechanisms to systems and sustainability (for expediency, we treat biomimicry as synonymous with bioinspired design, biologically inspired design, or biomimetic design [1,2,3], although this is not in line with ISO 18458 [4]). While these possibilities exist, our teaching experience, like others’, suggests the practice tends towards insights around narrower natural forms [5,6]. Even though biomimicry affords the possibility to address sustainability [2,3,4,7], its current practice in various modes does not necessarily lead to doing so [3,4,6,8,9,10,11,12].

The difficulties associated with incorporating sustainability within biomimicry are numerous and varied [3,8,9,10,11]. We overwhelmingly saw instructional examples, reports, and case studies of biomimicry applied at the product or product-technology scope, and at a scope that is too narrow to address sustainability broadly. Practice within this scope appears to lead to acceptance of narrow constructions of sustainability, such as increased efficiency [9,13]. In addition, while a few forms of an efficiency-based perspective are genuinely systemic, many are arguably not [13,14,15,16]. This issue goes beyond pedagogy in which environmental impacts are posed as the challenge for a biomimicry project.

At one level, this prevailing outcome is understandable. Much of the guidance we found available for practicing biomimicry is communicated at the level of organismal natural models (despite being more broadly applicable) [11] and their various constituent forms and functions, whether it is for a Biology to Design (Solution Driven or Technology Push) or a Challenge to Biology (Problem Driven or Technology Pull) process [4,5,6,17]. Information that is cataloged and accessible to practitioners appears to be at the organismal level, although not true of all resources [18,19]. This level seems to be the easiest to learn, share, and support. After all, individual organisms are concrete, distinct, approachable, remarkable beings imbued with character and wonder. Unfortunately, there is an ease with which organisms and products (or services) are associated, providing a narrow entrée into design-by-analogy thinking, biasing practitioners towards reductionist thinking and away from sustainability.

At another level, we accept the assertion that nature provides models for sustainability, and that there are abundant examples of natural systems and patterns to productively learn sustainability from, even if there are exceptions [4,5,17]. If this is indeed true, then we get what we ask nature for: the biomimicry practitioner’s intent must be for sustainability in order to get sustainability as an outcome [3,6,9,20]. Yet, most designers, our engineering students included, are situated at the product or product-technology scope, concerned with solving particular functional problems (the What) and not the general systems (the How) that produce functional artifacts [2,21,22]. The same must be true of the protagonists in many of the biomimicry stories told to socialize the practice. Expecting a holistic sustainability outcome from practitioners caught within conventional systems of production and consumption feels unreasonable. However, maintaining that relationship should not be acceptable.

Curricular studies are available describing the introduction of biomimicry in different ways across disciplines including architecture, business, design, engineering, and science among others, which are summarized elsewhere [17,23,24,25]. We did not find course reports or design guides that explicitly scaffold teaching sustainability as part of biomimicry practice. This situation is compounded by two additional issues. First, teaching and practicing biomimicry authentically is challenging and fraught with practical, methodological, and epistemological issues [2,5,9,25,26,27,28,29,30,31,32]. Second, there is a lack of knowledge of sustainability principles and practices on all sides. Most students and instructors new to biomimicry in our experience do not come with significant sustainability training. Even if they do, they vary, and it can be difficult to integrate sustainability concepts that did not originate within biomimicry, such as from life cycle analysis, ecodesign, cradle to cradle, green chemistry, or systems thinking [3,8,15,33,34,35,36,37,38], especially social ones [2,39].

This report is a response to the above observations. We contribute to an understanding of incorporating sustainability in biomimicry-oriented teaching and learning by describing our work over several years to adapt and integrate available biomimicry and design language, tools, and methods to go beyond structure-function to address conditions conducive to life directly in a biomimicry course for undergraduate engineering students. We developed a pedagogical approach and experience for teaching and learning sustainability within biomimicry practice. There are many other sustainability methodologies, e.g., life cycle assessment [2,35]. Doing so allowed us to engage design- and biology-minded students in an innovation process addressing ethical concerns and responsibility to society and the natural world, which we take to be imperative. We describe our efforts to create scaffolds, so novice learners ultimately reach a level of biomimicry practice that explicitly acknowledges and integrates sustainability.

## 2. Pedagogical Framing

**Figure 1:**
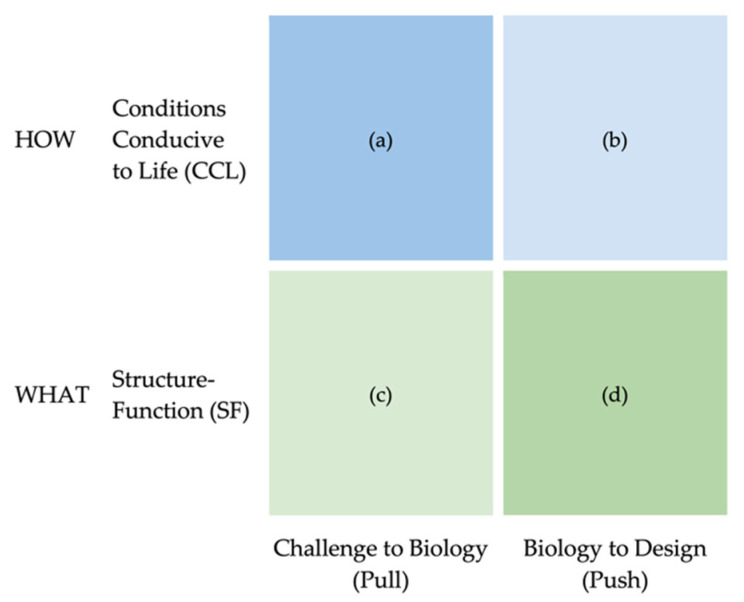
A view of biomimicry practice consisting of processes that span both the Structure-Function (**c**,**d**) aspects to be learned from nature (What nature does) and the Conditions Conducive to Life (**a**,**b**) aspects that speak to sustainability (How nature does What it does).

We made choices to frame curricular material conceptually to throw sustainability within biomimicry practice into sharp relief. We named and articulated two perspectives or points of view using terms common in biology (Figure 1). The first we termed Structure-Function (SF), represents the practice of learning from natural forms, processes, and ecosystems in the sense of specifying embodiment (the What), with learning from form to address function likely the most common. This choice is in line with the common use of these terms within biology. The second follows another existing conception, “life creates conditions conducive to life” or simply Conditions Conducive to Life (CCL) [6,20], representing learning from natural processes and ecosystems to address sustainability [20] in the sense of condition or quality (the How). These terms work well in natural language describing design practice. For example, “What is the structure-function relationship you are mimicking?” and “How are you creating conditions conducive to life?” We also explicitly delineated the Nature and Society domains, creating space for students to address the social construction of challenges and subsequent applications of abstracted design principles [3,6], in our case using inclusive participatory design methodology.

Naming and making the sustainability perspective (CCL) explicit and distinct made it more possible to indicate that just because a designer or design team was practicing one did not mean they were automatically practicing the other; that there are relative amounts of intention for and attention to each perspective in a given process. Furthermore, while one would have to take the SF perspective to gain insight on solving a particular design challenge (figure out the What), it would be potentially irresponsible or unethical to not also take the CCL perspective for the same challenge (figure out the How), a point that is otherwise challenging to convey without naming this perspective.

We chose to take Nature’s Unifying Patterns [19] as one holistic description of sustainable natural systems. These patterns are akin to design heuristics making them accessible to and practical for designers to use; heuristics by definition are not expected to be perfect, unique, nor complete, rather open to revision based on experience with use. We found the patterns to be more challenge and artifact-oriented and well suited to Challenge-to-Biology processes. We also used Life’s Principles [6,8,20,25,40], which are well suited to a Biology-to-Design process. Students benefit from structured exploration of Life’s Principles to increase familiarity [25].

The patterns can be used as a generative device (such as during ideation) or a screening device (akin to customer requirements screening in concept selection) [6]. Considerable SF work must be done before a CCL screen can be applied, necessitating multiple iterations that can be hard to find the project time for in a one-semester course. Taking the screening approach would be challenging to novice designers as it requires more discipline to maintain ambiguity and an ability to “kill all your (SF) darlings” (attributed to William Faulkner). Furthermore, screening may cause anchoring or fixation on SF approaches [5,41,42], something we are trying to avoid. We opted for the generative approach in the classroom context given our limited timeframe allowing us to make the CCL perspective central at the beginning of a design project.

A fundamental difficulty with natural processes and ecosystems as a guide for the sustainability of human systems is that Nature’s Unifying Patterns are all in effect simultaneously in nature. Addressing all of them simultaneously is a daunting consideration for the designer. Thus, when the patterns are used generatively, their collective consideration can lead to formidable search for and synthesis of insights from biological models if done reductively. This points to the need for designers to work within production and consumption systems that support and reinforce these patterns, and more broadly to new roles for designers to realize these more sustainable systems [11].

## 3. Conceptual Scaffolding

**Figure 2:**
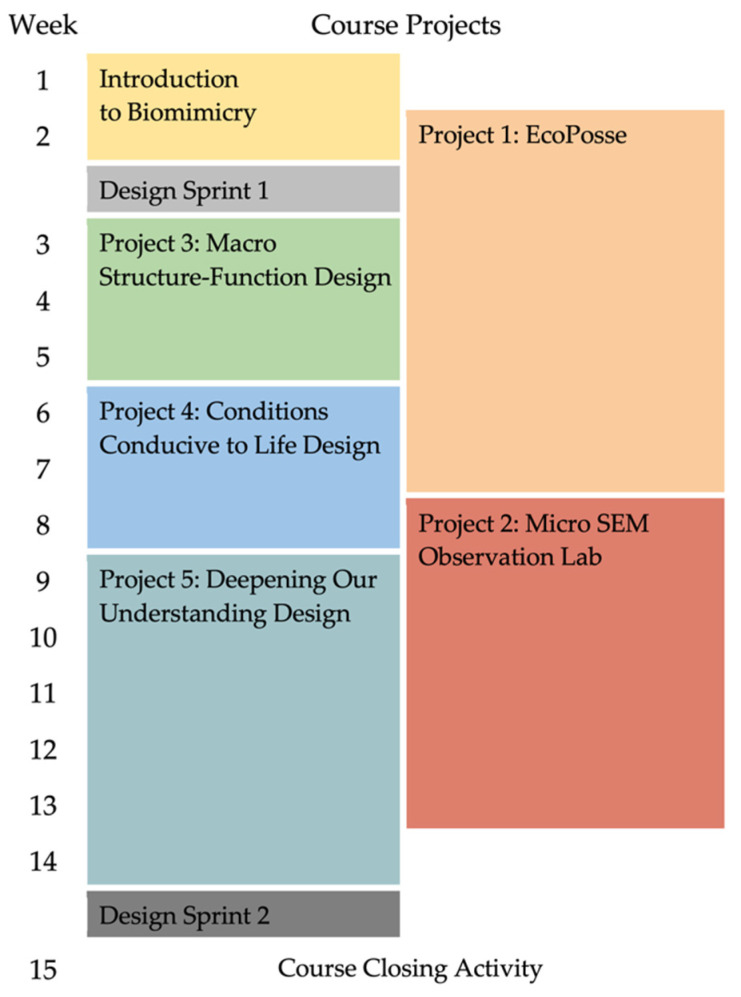
Course project schedule. See Appendix A for project descriptions.

Given the conceptual challenges described and drawing from our own experiences grappling with the uniqueness and complexity of biomimicry practice, we broke down a full SF plus CCL design process into manageable, progressive sets of design moves to conceptually scaffold [43,44,45] the learning experience for novice biomimicry students. The resulting experience consisted of three design projects in a 14-week semester, shown as Project 3 (individual), Project 4 (individual), and Project 5 (team-based) in Figure 2. Projects 1 (individual) and 2 (team-based) focused on building knowledge of the natural world and practicing observation skills and are included for completeness.

This sequence of projects was designed to facilitate practice with skills of observation, translation, and application, working with SF and CCL separately first, and then together in the final project. Project 3 is a shorter, more approachable, Biology-to-Design learning experience [46] addressing solely the SF perspective to initiate students into biomimicry, see quadrant in Figure 1d. Project 4 is a novel Challenge-to-Biology experience addressing only the CCL perspective to bring an explicit, intentional emphasis to sustainability, see quadrant Figure 1a, and elaborated below. In Project 5, the final project, students take on a full Challenge-to-Biology-to-Design process addressing SF and CCL together (Figure 1a–d). The emphasis on teaching CCL explicitly (design projects located in Figure 1a or b separately or in combination with c and d) differs from other approaches [17,23,24] that almost always teach SF alone (design projects located in Figure 1c or d separately or in combination), often assuming sustainability is being addressed implicitly [2,4,25].

In Project 4, which focused solely on exploring the CCL perspective, each student started with the general, systemic challenge of “creating products and services sustainably”. Conventionally, designers would start a Challenge-to-Biology process by identifying a concrete challenge or problem and then abstracting the desired functions that a design or solution would have to provide to meet the identified need. Analogously for this project, each team chose one of Nature’s Unifying Patterns as an already-abstracted function of a desired sustainable system of production and consumption. From here, students generated biologized questions [6] based on their adopted Unifying Pattern and created variations by considering relevant Life’s Principles [6] and using reframing design techniques [47]. After finding natural models and their associated contexts, students abstracted biological mechanisms and de-biologized them to articulate design principles informing the original patterns. Note the biologized question technique uses the form “How would nature…?” [6], a technique that applies to both the How (SF) and What (CCL) perspectives, involving different uses of “how”.

## 4. Methods

We assessed the development of student thinking within the biomimicry design process through a pre- and post-course design sprint activity that occurred in the second and then again, in identical form, in the 14th weeks of the course. The design sprints occur before and after three course projects with one, Project 4, being an explicit focus on understanding of CCL concepts (Figure 2). Observations from project outcomes from Project 3 and Project 5 that flank Project 4 additionally inform our assessment of pedagogical effectiveness for incorporation of CCL into the students’ biomimicry design process. For the first step in this activity, students were asked to explore the conceptual space around a challenge: “How might we create personal flight for individuals inspired by nature, and how might we do so sustainably?” Students were prompted to list several questions completed in the format of “How would nature…?” where students would indicate their approach to seek insight from nature to inform the challenge. The student-generated questions were independently scored by research staff with no knowledge of the study design according to whether they predominantly indicated structure-function (SF) considerations or Conditions Conducive to Life (CCL) considerations. SF considerations were those that focused on form and associated functions that exist in nature, e.g., structures that enable flight in different organisms. CCL considerations were those that examined processes that nature carries out that affect sustainability, e.g., process level considerations and principles as outlined by Nature’s Unifying Patterns. The Mann–Whitney U test was performed to determine statistical significance of CCL and SF considerations in the students’ biomimicry design process assessed by Design Sprint 1 and 2 that were pre and post the Project 4 CCL course pedagogy intervention.

## 5. Results

### 5.1. Pre- and Post-Design Sprint Insights

Students generated a variety of biologized challenge questions to address the design sprint prompt of sustainable flight. Example questions and SF or CCL classifications are listed in Table 1.

**Figure 3:**
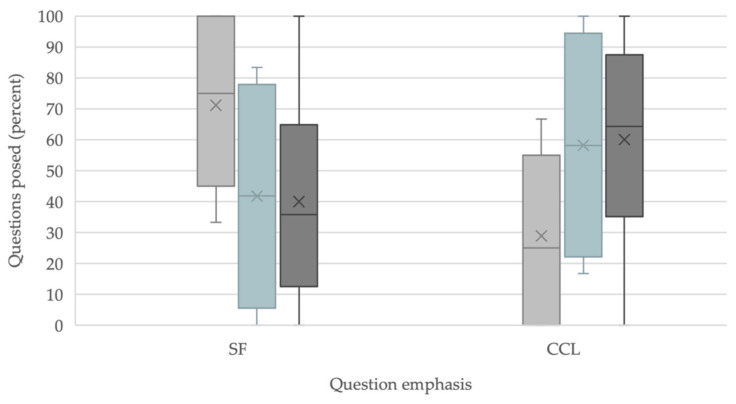
Percentage of questions that were considerations of SF or CCL from Design Sprint 1 (light gray bars), Project 5 (turquoise bar), and Design Sprint 2 (dark gray bars). The differences between percentages of CCL and SF questions asked were significant (Mann–Whitney U for CCL: *p* = 0.002, Z = −3.10, U = 41, and for SF: *p* = 0.010, Z = −2.56, U = 54.5).

**Figure 4:**
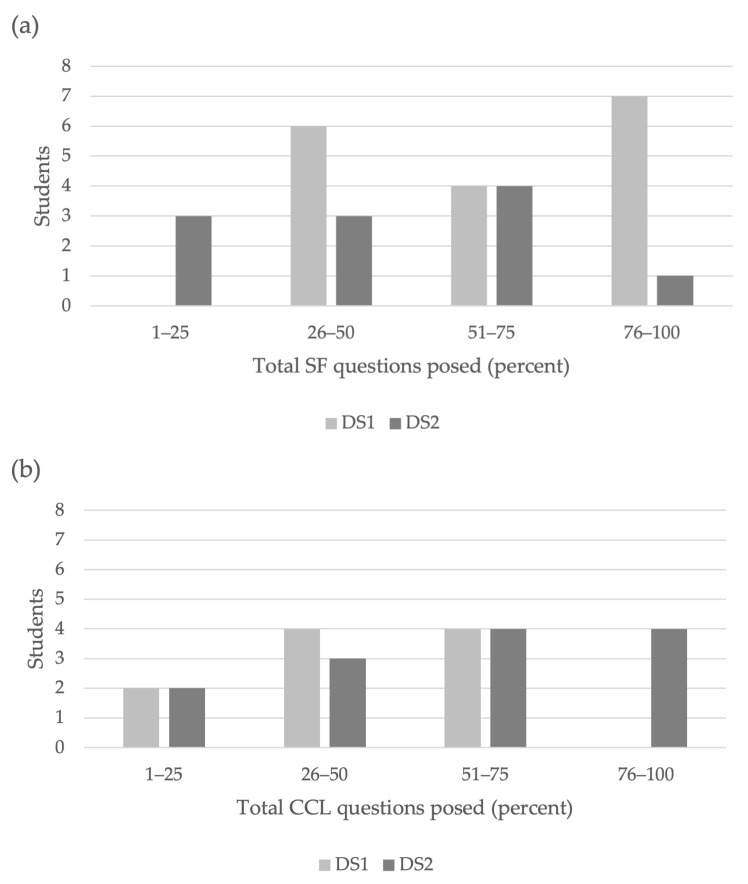
Numbers of students and percentages of their total questions that were (**a**) SF considerations or (**b**) CCL considerations in Design Sprint 1 (DS1), which took place during the second week of class, and Design Sprint 2 (DS2), which took place during the 14th week of class. Both design sprints had greater than 85% class participation. Sixteen students completed the class.

There was a significant difference in student generated design sprint questions that were classified as having CCL consideration as a percentage of all questions asked (Mann–Whitney U, *p* = 0.002, Figure 3) and an increase in percentage of students who generated CCL-related questions that were >50% of their questions asked (Figure 4) from Design Sprint 1 to Design Sprint 2, indicating that students developed greater consideration for CCL over the time of the course and integrated CCL thinking into their biomimicry design choices. The percentage of CCL questions that students asked for their final Project 5 additionally resembled those in Design Sprint 2 (Figure 3), indicating that student consideration of CCL in their ways of thinking in the field persisted across multiple class activities to the end of the course.

At the start of the course, greater than half (11/16) of all students posed questions that were mainly (>50%) SF in consideration (Figure 4a). This may reflect novice learners’ exposure to and ways of thinking about biomimicry as SF considerations are more straight-forward to understand as described earlier. At the course end, student thinking shows incorporation of CCL and Nature’s Patterns into their biomimicry design choices as evidenced by the increase in CCL questions that students proposed in their searches of nature in Project 5 (Figure 3), elaborated in the Observations section below, and a shift of the majority of students posing CCL questions from the pre- and post-design sprints (Figure 4b). This is likely due to the influence of course pedagogies that were employed.

### 5.2. Observations and Outcomes from Student Projects

With the initial Project 3, where students translated biological structure, function, and mechanism into design principles and applications, the translations resulted in applications that were based mostly on form and were predominantly literal. For example, student applications for projects included a filtration system for microplastics based on the form of the manta ray gill that is used for filter feeding and a retractable tire traction system based on leopard claws that retract into the paw. These literal translations based on form may be more accessible to students that are new to biomimicry and to the translation that is required between fields [5].

We [6,40] introduced Project 4 as a scaffold to explicitly focus on understanding of CCL concepts by engaging students in examination of functions and mechanisms by which nature accomplishes CCL. Students developed biologized challenge questions based on systemic patterns and principles of nature and analyzed natural models for sustainability insights. The dedicated study of CCL resulted in project outcomes and discussion about how natural models embody multiple unifying patterns and principles and that not all natural functions necessarily result in CCL. For example, different student groups studying CCL principles of cooperation and resiliency both examined lichen as a natural model. For cooperation, students highlighted biological strategies of how algae and fungi exchange resources, how nature evolved to become interdependent and achieve a task outside of a single organism’s capability, and trade-offs in cooperation between organisms and structural relationships. With respect to resilience, another group highlighted strategies for the robustness of lichen to environmental changes: the structural aspects of drought tolerance, nutrient acquisition, dehydration mechanisms, and decentralized reproductive strategy. Students observed through design presentations with gallery sketches of natural models that the other team’s analysis of lichen highlighted different mechanisms regarding CCL. Student reflections from this project demonstrated understanding of CCL insights and the viewing of natural models and structure-function in light of CCL.

In the final project (Project 5), students demonstrated integration of SF and CCL considerations in their design outcomes. For example, in studying challenges related to urban sprawl for this project, one student team made connections between how reef morphology facilitates a network of cooperative species, and in their proposed solution, similarly designed for diversity and community within living spaces in cities. The CCL insight was that cohabitation as seen in nature utilizes diverse strengths of a community that can be emulated. Students studying a challenge in architecture gained inspiration from niche differentiation by looking at ways that humans could utilize lesser occupied niches in nature and develop alternative means and structures for human living. Students looked to natural models for examples of how to live, and they applied a CCL principle when they took inspiration to search for solutions for human living that fulfilled a different niche in an ecosystem. There was evidence of divergent thinking and viewing of nature as mentor [5] and more nuanced insights from natural models.

## 6. Discussion

In this study, we found students integrated CCL thinking into their biomimicry design choices, based on pre- and post-design sprint results and evidenced by student design project outcomes. Students posited a significantly greater percentage of CCL-related biologized challenge questions from Design Sprint 1 to Design Sprint 2 and demonstrated CCL considerations in final project designs. However, the sample size was small due to the available class size and all students were provided the pedagogical sustainability intervention obviating a control group.

The design project outcomes we observed contribute qualitative evidence to aid the interpretation and understanding of the design sprint results. Though Nature’s Unifying Patterns and Life’s Principles [6,40] were provided to students and referenced early in the course, these CCL considerations did not become incorporated into student design outcomes in the Biology-to-Design project (Project 3). The Project 4 focus on deep understanding of Nature’s Unifying Patterns and associated activities emphasized the distinctions between SF and sustainability insights. We observed the evolution of student skills in Project 5 following the Project 3 and Project 4 scaffolding. With practice, students improved their skills for viewing the natural world and understanding of natural processes and environmental context. Student thinking also tended towards more metaphorical and nuanced translations over time, including CCL considerations as supported by Design Sprint 1, Design Sprint 2, and Project 5 data.

How students viewed biological systems, their thought processes, and solutions in the final project (Project 5) demonstrated integration of SF and CCL components. The integrated CCL components in Project 5 as compared to Project 3 suggest that scaffolded learning of CCL in Project 4 enabled student understanding and internalization of CCL in practice. Thus, the aim of the course for students to include consideration of CCL in a complex design process was met.

## 7. Conclusions

While educators and facilitators have options for addressing sustainability, few techniques have been shared for emphasizing CCL practice in the classroom setting or with novice practitioners. We developed a pedagogical framing and conceptual scaffolding that allowed us to incorporate sustainability into a college level biomimicry learning experience for engineering students, drawing extensively on existing pedagogical resources. This approach elevates and puts CCL on equal grounding to SF, and pedagogically, learning activities that solely focus on CCL practice are essential to providing the experience students need to incorporate this view.

We are encouraged by signs of creative systemic thinking and higher-level learning from nature and a discernible shift away from a narrower structure-function practice towards addressing conditions that are conducive to life.

Because broader sustainability practice within biomimicry remains largely unaddressed, because biomimicry educators are initiators of a turn to nature and a biologically endowed practice, all biomimicry educators and facilitators should individually and collectively commit to always include an explicit sustainability approach within their pedagogy [48,49,50], or, at a minimum, explicitly acknowledge to their participants that the practice does not provide for it necessarily. Taking on this commitment would help us collectively search for and shift to practices that are conducive to life.

## Figures and Tables

**Table 1:** Student design sprint question response examples and Structure-Function (SF) and Conditions Conducive to Life (CCL) classifications.

Example Questions
**SF**
How would nature travel long distances in the air?
How does nature reduce drag?
How would nature allow for a safe landing?
How does nature do quick rapid flight?
**CCL**
How does nature use readily available resources to fly?
How does nature conserve energy while flying/swimming?
How would nature use natural materials and chemistry for flight?
How would nature use naturally occurring elements of weather to generate lift?

## Data Availability

Data associated with this study are available upon request.

## Appendix A

### Project Descriptions

Project 1: EcoPosse. Individual students selected an inspirational group of organisms, or EcoPosse by researching, reflecting upon, and selecting 6–8 natural models (at least 2 organisms each exemplifying levels of Structure-Function through Form, 2 organisms exemplifying Process, and 2 biological communities, habitats, or biotopes exemplifying Ecosystem). Students collected information about each posse member that they selected in profile pages using a template provided for either organisms or ecosystems. Students then developed a 1-slide graphical representation of their selected group of organisms, ecosystem, and processes and shared highlights and unique characteristics of their EcoPosse as a 3-min presentation to the class.

Project 2: Micro SEM Observation Lab. Students designed and conducted experiments to examine the natural world using a scanning electron microscope (SEM). The goal of this project was to expand student experience working with and making observations from nature at the micro scale. Examples of projects were comparative studies of feathers, of butterfly wings that exhibited structural color and those that did not, and of needle structures from cacti and porcupines. The goals of the initial Projects 1 and 2 were for students to gain experience with analysis of biological systems and an appreciation of natural models. Observational and hands-on assignments (outdoor, video, and artifacts observations) and guided reading and discussion of primary literature in biology continued throughout the term to encourage development of connections to natural systems beyond the surface level.

Project 3: Structure-Function Design. Individual students started with identification and examination of a natural model (observation), described the biological mechanisms displayed by the model, abstracted the mechanism to generalizable design principles (translation), and, in the last stage, developed potential applications for the design insights (application). This project was an accessible first project for students in biomimicry. Examples of projects were a juice concentrator inspired by the kangaroo rat kidney, packaging inspired by fungal spore structures, and soft robotic handling inspired by raptor beaks.

Project 4: Conditions Conducive to Life Design. Individual students specifically considered and examined CCL processes and how nature accomplishes CCL. The starting point of the project was sustainability as the goal: what insights can nature provide for how humans can live more sustainably? With this as the starting point (application), students selected one of Nature’s Unifying Patterns [6], developed biologized challenge questions based on their pattern (translation), and investigated natural models that carried out these processes (observation). From these natural models, the students then identified design principles (translation) and reflected on these design insights drawn from nature about how humans could live more sustainably. Having a design activity focused on CCL had the goal of putting sustainability at the forefront and providing students practice in viewing structure-function from natural systems through CCL criteria.

Project 5: Deepening our Understanding Design. combined design processes of Projects 3 and 4. Students initiated the biomimicry process at the stage of application by defining challenges from a subset of broad application areas brainstormed by the class (e.g., urban planning, architecture, commerce, agriculture, disaster response). Teams of students carried out research to define specific challenges within these application areas with attention to stakeholders. They then approached solutions to these challenges using biomimicry by engaging in SF and CCL across the process steps from Projects 3 and 4.
